# The chitin raft hypothesis for the colonization of the open ocean by cyanobacteria

**DOI:** 10.1098/rstb.2024.0086

**Published:** 2025-08-07

**Authors:** Rogier Braakman

**Affiliations:** ^1^ Department of Earth, Atmospheric and Planetary Sciences, Massachusetts Institute of Technology, Cambridge, MA, USA

**Keywords:** planktonic marine cyanobacteria, metabolic evolution, biospheric productivity, atmospheric oxygenation

## Abstract

It is often assumed planktonic cyanobacteria existed in Precambrian oceans, but that their productivity was constrained. However, available evidence suggests picocyanobacteria only colonized the open ocean near the Neoproterozoic–Phanerozoic boundary, close to the start of a period of sustained atmospheric oxygenation. If earlier open oceans were devoid of planktonic cyanobacteria, we lack consensus explanations for why this was the case. Colleagues and I recently introduced the ‘chitin raft hypothesis’, which argues that accumulating chitin particulate waste associated with the rise of arthropods provided an essential evolutionary stepping stone in the rise of marine picocyanobacteria. According to this hypothesis, chitin particles derived from arthropod exoskeleton moults offered marine picocyanobacteria refugia from environmental stresses in the water column, allowing them to explore—and begin adapting to—the open ocean for the first time. Here, I review the context and implications of this hypothesis. One implication is that Precambrian biospheric productivity was constrained by the total global volume of benthic habitats. Hence, the rise of sub-aerial continents near the Archaean–Proterozoic boundary would have driven a major increase in biospheric productivity, with the expansion of oxygenic photosynthesis into the open ocean and onto the continents near the Proterozoic–Phanerozoic boundary driving a second major increase.

This article is part of the discussion meeting issue ‘Chance and purpose in the evolution of biospheres’.

## 1. Introduction

The oxygenation of Earth’s atmosphere resulted from an interplay of multiple biological and geological processes but was ultimately driven by the photosynthetic production of oxygen and the long-term sequestration of associated organic carbon in Earth’s mantle, crust and oceans [1,2]. Understanding large-scale patterns in biospheric productivity over Earth’s history [3] is key to reconstructing the rise of oxygen in Earth’s atmosphere. Biospheric productivity is determined by the size and spatial extent of different ecosystems in which oxygenic photosynthesis occurs and the rate of photosynthesis in those ecosystems. Both aspects are challenging to precisely constrain in reconstructions of early Earth environments as they depend on inferences from geochemical proxies and fossil/biomarker records that become sparser and more ambiguous in deep time, especially for the deep open ocean [4–6]. This in turn impacts molecular clock calculations of the evolution of major groups of photosynthetic organisms, which depend on external constraints from the geologic record [5,7,8]. Together this leads to generally greater uncertainty regarding the scale of primary production when extrapolating further back in Earth’s history.

Studies of microbial metabolism and physiology can help address these uncertainties by suggesting linkages between the geologic and genomic records. I will illustrate this using marine picocyanobacteria, consisting of the sister lineages *Prochlorococcus* [9] and marine *Synechococcus* [10]. Marine picocyanobacteria perform approximately 20–25% of CO_2_ fixation in extant oceans [11,12] and their rise may have been linked to general biospheric revolutions of the late Proterozoic (2500–541 Ma) and early Phanerozoic (541 Ma to present) that ultimately led to the rise of macroscopic eukaryotic life and complex trophic ecosystems [13–15]. As background I will first review geologically informed frameworks that have been proposed for the productivity of the marine biosphere during the Proterozoic, as well as evidence regarding the timelines of evolution of planktonic marine cyanobacteria in the open ocean. I will then review the chitin raft hypothesis, a macroevolutionary framework we recently proposed for how marine picocyanobacteria colonized the open ocean, and end by discussing implications of this hypothesis for large-scale patterns of biospheric productivity over Earth’s history.

## 2. Biospheric productivity in Proterozoic oceans

**Figure 1.**
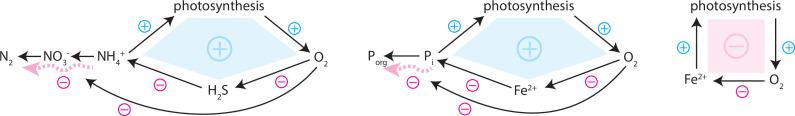
Previously proposed constraints on productivity of the ocean biosphere during the Proterozoic. Blue positive symbols reflect positive, stimulatory effects, while pink negative symbols reflect negative, inhibitory effects. Availability of nutrients (nitrogen, phosphorus, iron) stimulates photosynthesis and thereby O_2_ production. Nitrogen and phosphorus availability are proposed to have been limited due to chemical feedbacks, as well as due to differential rates within the biological cycles of both elements, the latter represented as dashed pink arrows. Increased O_2_ levels would have helped eliminate proposed negative feedbacks, leading to overall self-amplifying cycles (blue shading) that previously may have been stuck in local minima. In contrast, iron availability is proposed to have *become* limiting under rising O_2_ levels due to its decreasing solubility, leading to an overall self-damping cycle (pink shading). See the text for further details. Note: phytoplankton can use multiple forms of nitrogen, but the universal entry point of nitrogen into metabolism for all life is ammonia. P_i_, inorganic phosphorus (i.e. phosphate); P_org_, organic phosphorus.

Atmospheric oxygen underwent a dramatic rise in the early Proterozoic [16–18], but then remained low relative to modern levels until a second dramatic rise occurred between the late Proterozoic and early Phanerozoic [19–21]. Various constraints on productivity of the Proterozoic marine biosphere have been proposed (figure 1), several of which involve the interplay between metals and the macronutrients nitrogen and phosphorus. For example, it has been suggested that molybdenum and other metals essential to the biological nitrogen cycle would have been scavenged by sulfide ions in sulfide-rich (i.e. euxinic) waters, leading to nitrogen limitation of photosynthesis [22,23]. Euxinic conditions were likely limited to relatively productive coastal waters, but models suggest that such environments could potentially still have acted as an effective global sink of molybdenum due to large-scale ocean circulation processes [23]. Similarly, it has been suggested that the high levels of ferrous iron (Fe^2+^) in the mostly anoxic Proterozoic oceans [24,25] would have scavenged free phosphate, leading to phosphorus limitation of photosynthesis [26,27].

Other proposals focus on limitations arising due to differences in the rates of processes within the biological cycles of nitrogen and phosphorus under conditions of low oxygen (figure 1). That is, when oxygen is low, denitrification (i.e. conversion of NO_3_^−^ to N_2_ gas) rates are predicted to outpace nitrification (i.e. conversion of NH_4_^+^ to NO_3_^−^), preventing NO_3_^−^ from accumulating, thereby driving nitrogen loss (i.e. outgassing of N_2_ from the ocean) and nitrogen limitation [28]. Similarly, when oxygen is low, remineralization of organic phosphate to inorganic phosphate is predicted to have been suppressed, driving or exacerbating phosphorus limitation [29].

These proposals share two common properties. One is that a rise in oxygen towards Phanerozoic levels ultimately acts to remove preceding nutrient limitations (figure 1). That is, oxygenating the ocean removes both sulfides and ferrous iron, thereby eliminating the scavenging of molybdenum [30] and phosphorus [31], respectively. Simultaneously, a rise in oxygen levels increases the rate of phosphorus remineralization [29] and the rate of nitrification relative to denitrification, thereby counteracting nitrogen loss [28] (figure 1). Indeed, relative to Proterozoic sediments, Phanerozoic sediments show an increase in the levels of molybdenum [30] and phosphorus [27,31], as well as the onset of a stable oceanic nitrate pool [32,33], consistent with these proposals. However, it should be noted that most of the geochemical records used to make inferences of ocean nutrient inventories are derived from sediments deposited in shallow continental shelf environments [27,30–33], leading to inevitable uncertainties regarding the nutrient landscape of the open ocean. Still, molecular clocks suggest that nitrogen-fixing marine planktonic cyanobacteria did not expand into the open ocean until the late Neoproterozoic or early Phanerozoic [34], consistent with nutrient-poor (or at least nitrogen-poor) oceans before this time.

The second common feature of these proposals is that they generally assume that planktonic cyanobacteria were present in the open ocean, but with their productivity suppressed due to the proposed nutrient limitations. These combined features create the potential for nonlinear dynamics in the oxygenation of the ocean and atmosphere. That is, increases in oxygen will be transient so long as the marine biosphere remains in the grip of outlined nutrient limitations but can become self-sustaining if they are significant enough to begin loosening constraints on the productivity of pre-existing planktonic cyanobacteria (figure 1) [35]. This has led to the argument that a large-scale redox perturbation due to Snowball Earth played a key role in triggering the transition to an oxygenated ocean by driving a temporary increase in phosphorus availability that ultimately became self-sustaining [35]. Proposals that seek to explain the time course of atmospheric oxygenation through long-term changes in geologic processes rather than through changes in constraints on biospheric productivity often similarly assume, either implicitly or explicitly, that cyanobacteria were present in the open ocean [20,36–38]. However, the validity of the assumption that planktonic cyanobacteria existed in the open ocean in the Proterozoic is not clear.

A different dynamic emerges in a proposal that colleagues and I put forward [39,40], in which productivity of Proterozoic oceans was limited at least in part due to a negative feedback built into the molecular machinery of photosynthesis itself (figure 1). That is, photosynthesis depends on iron, but iron is insoluble in the presence of oxygen. Thus, as oxygenation proceeds, iron becomes less available, lowering the production of oxygen, creating a self-damping dynamic that contrasts with the ultimately self-amplifying dynamics as in the proposals outlined above (figure 1). Indeed, this proposed dynamic is independent of the ocean inventories of nitrogen and phosphorus, suggesting that external perturbations [35] or long-term changes in geologic processes [20,36–38] were insufficient to drive oxygenation of the open ocean and that biological innovations were necessary to unlock the full productivity potential of the ocean [39–41]. If correct, this raises the question of what triggered these innovations.

## 3. Reconstructed timelines for the evolution of planktonic marine cyanobacteria

Evaluating proposals and models for marine biospheric productivity in deep time requires considering the evidence for the existence of planktonic marine cyanobacteria in the open ocean throughout Earth’s history. Indirect evidence comes from observations regarding the geochemical record of carbon in two ways. The first is the high organic carbon content of marine shales reaching back as far as the Archaean (4.0–2.5 Ga), which has been argued to require the presence of planktonic cyanobacteria to allow the production of observed levels of organic carbon (e.g. [42]). However, marine shales are mostly derived from shallow water environments that may not be reflective of the open ocean, and benthic mats can reach very high levels of production [43], especially in Precambrian oceans when grazers capable of effectively breaking up microbial mats were largely absent [44,45].

A second source of indirect evidence comes from the geologic record of carbon isotopes, which shows nearshore-to-offshore gradients in the ^13^C content of sedimentary carbonates that could be consistent with export (and subsequent remineralization) of organic carbon from surface waters into sediments in the Proterozoic [46] and Archaean [47]. However, observed gradients are weak, and conclusions regarding export processes thereby depend on assumptions that gradients are muted due to the high levels of atmospheric CO_2_ [46,47] needed to maintain liquid oceans on the early Earth due to lower solar luminosity [48]. Precise levels of CO_2_ relative to other potential greenhouse gases (particularly CH_4_) in deep time remain debated [49,50], leaving uncertainty about the magnitude of potential export processes. Moreover, offshore isotopic records are limited in number and typically come from depositional environments that extend at most a few tens of kilometres beyond the shelf break [46,47]. In modern oceans, continental shelves and near-shore open ocean waters are connected through physical transport processes driven by tides, wind stress and general ocean circulation [51,52]. This leaves open the possibility that observed carbon isotopic gradients could have been generated by primary production within continental shelves followed by lateral transport of organic carbon to deeper waters.

Direct evidence specific to the evolution of cyanobacteria ultimately comes from two sources: fossils and biomarkers available in ancient rocks [4–6,53], and the record of molecular evolution available in genomes of extant cyanobacteria [5,8,13]. The fossil record is often less definitive for cyanobacteria than for eukaryotes due to their smaller size and simpler structural morphologies [54,55]. Targeted genome sequencing efforts (e.g. [56]) and biomarker analyses (e.g. [57]) have increased the number of structural morphologies in rocks unambiguously tied to cyanobacterial taxa, but the numbers remain limited, and all are from shallow marine settings [4–6]. Recently, new fossils have been described that identified thylakoid-bearing cyanobacteria in 1.78−1.73 Ga and younger deposits [58] and branching filamentous N_2_-fixing cyanobacteria (order Nostocales) in 1.04−1.0 Ga deposits [59], both from benthic marine environments. These fossils add key temporal constraints on important branch points within the cyanobacterial tree. However, their morphologies—and indeed the morphologies of all recognized cyanobacteria fossils in general—are not characteristic of the smaller spherical or coccoidal individual cells of cyanobacteria with a dedicated planktonic lifestyle in open ocean waters [4–6,9,10].

Biomarkers in turn suggest that Proterozoic marine environments were dominated by cyanobacterial photosynthesis [53]. However, while the isotopic shifts of these molecules are compatible with those from planktonic cyanobacteria, their taxonomic assignment remains uncertain [53] and so similarly cannot distinguish between groups with a dedicated planktonic lifestyle and those that can switch between surface attached and planktonic states. Thus, taken together, fossil and biomarker records from Proterozoic sediments are compatible with the presence of cyanobacteria capable of undergoing lifecycles involving both sedimentary or surface attached and planktonic phases in the benthic environment but do not offer evidence of planktonic cyanobacteria in the euphotic zone of the deep open ocean [4–6]. Whether this reflects inherent biases of the geologic record or a genuine absence of planktonic cells in the open ocean in deep time is an open question.

The genomic record, in turn, is clearer. Most molecular clock calculations suggest that ancestors of marine picocyanobacteria colonized the ocean in the late Neoproterozoic (1000–541 Ma) or early Phanerozoic [8,13,60–62], although origins in the early Neoproterozoic have also been found [63]. In general, molecular clock calculations of divergence times and associated uncertainty ranges are strongly dependent on the external constraints that are used and, as outlined above, the number of constraints available from the geologic record are very limited for cyanobacteria [4–6]. To help address this gap, a recently emerging approach is to use horizontal gene transfer (HGT) events as an additional source of constraint on molecular clock calculations [64]. That is, if the topologies of both the donor and recipient trees within the phylogenies of genes undergoing HGT are sufficiently similar to those of the species trees of both groups, and either group has an absolute date constraint, this constraint can be propagated across both groups [64]. Using this approach to calculate evolutionary timelines of cyanobacteria leads to a calculated mean age of 424 Ma (range 491–340 Ma) for crown group marine picocyanobacteria and a calculated mean age of 576 Ma (range 666–492 Ma) for ‘total group’ picocyanobacteria, the latter of which includes groups from freshwater and brackish environments [8]. These ages are consistent with other recent calculations [60,65] but reduce calculated uncertainties by several hundred million years. In other words, molecular clocks suggest that the cyanobacterial branch leading to crown group marine picocyanobacteria evolved over an approximately 150 Myr period that came after the last Neoproterozoic Snowball Earth (i.e. the Marinoan glaciation), which ended approximately 632 Ma [66]. These results are consistent with recently described fossils of thylakoid-bearing [58] and branching filamentous cyanobacteria [59], as the molecular clock calculations in Fournier *et al.* [8] suggested divergences of thylakoid-bearing cyanobacteria more than 2 Ga and of order Nostocales more than 1 Ga, both older than the minimum age constraints introduced by newly described fossils.

In summary, the genomic record suggests that ancestors of extant marine picocyanobacteria most likely colonized the open ocean in the early Phanerozoic, while the fossil and biomarker records offer no evidence of planktonic marine cyanobacteria in the pelagic realm before then. This leaves open two basic possibilities: (i) planktonic marine cyanobacteria *did* exist in the open ocean during the Precambrian but went extinct, possibly due to Snowball Earth glaciations, with evidence of their existence erased or yet to be discovered, or (ii) cyanobacteria colonized the open ocean for the first time in the early Phanerozoic. If the latter is true, why did cyanobacteria not colonize the ocean sooner? I will next discuss how the metabolism of extant marine picocyanobacteria provides relevant insights into these questions.

## 4. Emergence of planktonic marine picocyanobacteria: the ‘chitin raft hypothesis’

Microbial cellular metabolism is intertwined with chemical processes in the environment. Metabolic innovations that occur over microbial evolution therefore carry imprints of large-scale changes in the ecosystems these microbes are part of [39,40]. One such innovation that provides insight into the timing of the colonization of open ocean waters by cyanobacteria comes from a trait that colleagues and I recently discovered in extant marine picocyanobacteria: chitin degradation [67]. Chitin is an organic carbon polymer that is highly abundant in the ocean and is largely derived from the exoskeletons of arthropods [68]. The existence of genes for chitin degradation in marine picocyanobacteria is unexpected, as chitin exists mostly as particles, and marine picocyanobacteria are generally considered to live a planktonic lifestyle [13,69]. However, we showed that marine *Synechococcus* and deep-branching clades of *Prochlorococcus* attach to chitin particles and display enzymatic activity of chitinase, the main extracellular enzyme involved in cleaving chitin polymers into smaller units that cells can take up [67]. We also showed that expression of chitin degradation genes and other attachment-associated genes increases when cells are exposed to chitosan, a solubilized form of chitin, and that *Prochlorococcus* obtains a growth boost from exposure to chitin when grown at low light levels. These results demonstrate that chitin attachment and degradation is an active functional trait in marine picocyanobacteria, changing our view of their ecology. Some other cyanobacteria can use chitin, but in those cases have been shown to use it as a source of nitrogen [70], whereas marine picocyanobacteria do not respond to the presence of chitin in nitrogen-depleted media [67,70]. Instead, marine picocyanobacteria use chitin only as a supplemental carbon source under light-limited conditions [67], highlighting a general difference in the ecology of this trait in this group relative to other cyanobacteria.

**Figure 2.**
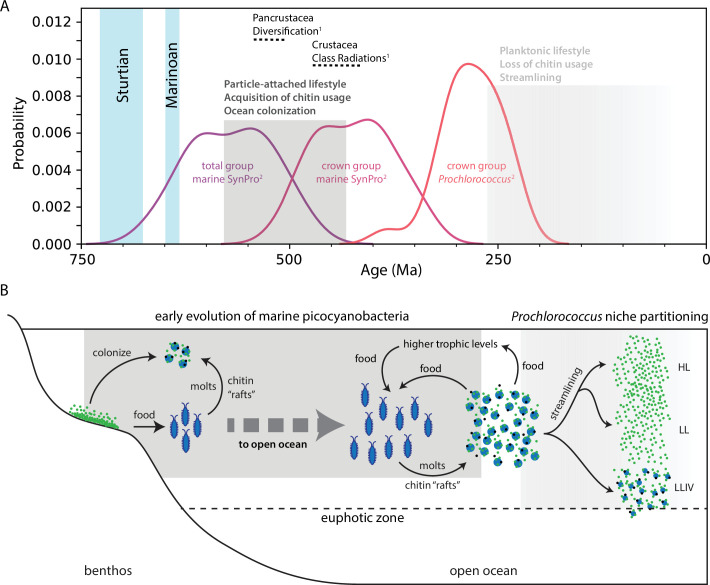
The chitin raft hypothesis. (A) Estimated timelines for the evolution of arthropods (^1^) [72] and marine picocyanobacteria (^2^) [8]. ‘Total group marine SynPro’ refers to where marine picocyanobacteria diverged from non-marine picocyanobacteria, whereas ‘crown group SynPro’ refers to the crown group marine picocyanobacteria, together defining the evolutionary branch along which the chitin usage trait was acquired. Vertical blue bars represent Snowball Earth glaciations that preceded the evolution of arthropods and marine picocyanobacteria. (B) Inferred ecological context of the chitin raft hypothesis. Marine picocyanobacteria and arthropods are proposed to have colonized the open ocean in tandem, in two stages. In the first stage, picocyanobacteria colonized chitin particles derived from the exoskeleton moults of arthropods, where aggregating on the surfaces of these particles allowed cells to collectively shelter from and counteract environmental stresses in the water column. Particle-associated picocyanobacteria in turn provided arthropods with a growing source of food in the water column, either directly or through transfer of carbon from primary production to higher trophic levels. This association is inferred to have allowed both groups to jointly expand out into the deep open ocean for the first time, thereby helping to seed the rise of modern marine ecosystems. Later, after acquiring adaptations to better withstand the stresses of life in the open ocean, some lineages of *Prochlorococcus* are proposed to have made a secondary evolutionary transition to a more dedicated planktonic lifestyle, with this transition being linked to a period of cellular and genomic streamlining. Notes: ‘HL’ and ‘LL’ refer to ‘high-light-adapted’ and ‘low-light-adapted’ clades of *Prochlorococcus*, respectively, that have lost the chitin usage trait and have undergone cellular and genomic streamlining. ‘LLIV’ in turn refers to the deeply branching, low-light-adapted IV clade of *Prochlorococcus*, which has larger cells and genomes and retains the chitin usage trait, giving this group access to a supplemental carbon and energy source under light-limiting conditions deep in the euphotic zone. Green cells represent marine picocyanobacteria and their ancestors, whereas black cells represent other microbes within particle-attached communities, from which marine picocyanobacteria ultimately obtained genes of the chitin usage trait. The figure is modified from Capovilla *et al.*[67].

How does the existence of chitin attachment and degradation capabilities in marine picocyanobacteria inform their macroevolution? First, combined phylogenetic and phylogenomic analyses indicate genes necessary for chitin degradation were acquired through HGT (likely from unspecified planctomycetes) along the branch leading from total group picocyanobacteria to crown group marine picocyanobacteria [67], which, as mentioned above, evolved over the period approximately 576–424 Ma [8]. Both the fossil record and molecular clocks in turn indicate that marine arthropods underwent a major ecological expansion starting around approximately 535–520 Ma [71–75]. This suggests that picocyanobacteria acquired the ability to use chitin over the same time frame that arthropod-derived chitin became increasingly available in the ocean (figure 2). Marine fungi are another important source of chitin, but their remains have been identified in fossils with ages of approximately 1 Ga [76], with molecular clocks placing the fungal crown group well before this [77], suggesting their evolution predates that of marine picocyanobacteria by 500 Myr or more. This temporal disconnect may reflect a key difference between chitin derived from arthropods and fungi. That is, whereas in arthropods, chitin is the major structural component of exoskeletons and is directly accessible at the surface [68,78], in fungi, chitin is tightly embedded with other materials in the inner portions of the cell wall [79,80], making it less directly accessible.

To further understand the relevance of the acquisition of the chitin attachment and degradation trait to the ecological rise of marine picocyanobacteria, it is instructive to consider microbial lifestyle in general. Global surveys suggest that outside of lakes and oceans, microbial biomass exists predominantly within aggregates attached to surfaces due to the collective stress buffering and nutrient recycling conditions such communities create [81]. Most microbes, including cyanobacteria, can facultatively switch between planktonic and aggregated states, and while there are no single universal stressors driving aggregation across all microbial systems, common triggers include salinity, UV radiation and nutrient stress [81–85], all of which are elevated in surface waters of the open ocean. Moreover, these stresses were likely exacerbated in the Precambrian, when UV levels were higher due to a less-developed ozone layer [86] and oceanic nutrient inventories may have been lower [22,23,26–34]. How then, did planktonic cyanobacteria colonize the open ocean in the face of stresses that generally favour aggregation?

In response to this question, we proposed the ‘chitin raft hypothesis’ (figure 2). In this hypothesis [67], accumulating chitin particles derived from the exoskeleton moults of arthropods provided a growing reservoir of surfaces where picocyanobacteria could aggregate and shelter from environmental stresses. Here, the idea of rafts is not meant to suggest that picocyanobacteria spread across the oceans on floating chitin particles, as indeed these particles generally sink rather than float [87], nor is it intended to reflect a direct relationship between picocyanobacteria and a living host. Rather, our hypothesis suggests that chitin particles derived from arthropod exoskeletons offered picocyanobacteria ‘refugia’ from the harsh environment of the open ocean, creating for the first time an opportunity to colonize it. In this view, the use of chitin as a material resource emerged later due to the transfer of genes from other microbes within particle-attached communities. This may also explain why the evolution of marine picocyanobacteria was specifically linked to the evolution of arthropods but not to the evolution of fungi or other chitin-containing organisms that do not supply chitin particles to the water column in the way, or at the rate, that moulting arthropods do. Growing populations of picocyanobacteria could in turn have provided a growing food source for arthropods in the open ocean, either directly or by fuelling ocean ecosystems from the bottom up through their primary production. Finally, the chitin raft hypothesis suggests that picocyanobacteria adapted to the open ocean over millions of years before some *Prochlorococcus* lineages made a secondary transition to a dedicated planktonic lifestyle [67].

The chitin raft hypothesis is consistent with several additional lines of evidence in marine picocyanobacteria. First, a growing number of associations of marine picocyanobacteria to particles and/or protist hosts have been identified in recent years [88,89], suggesting that surface attachments play a more significant role in the ecology and evolution of marine picocyanobacteria than historically recognized. Second, the adaptations seen in extant lineages include the acquisition of genes for compatible solutes that protect against osmotic stress from elevated salinity [90,91] and for protection from UV and light stress [92,93], as well as a series of cellular, macromolecular and metabolic innovations that lower the nutrient requirements of growth while raising nutrient affinity [39,94–97]. By helping cells adapt to the unique challenges of microbial life in the open ocean, these adaptations also mitigate against the stresses that are generally linked to cellular aggregation and surface attachment in microbes [81–85], thereby paving the way for the transition to a dedicated planktonic lifestyle in sub-groups of *Prochlorococcus*. Finally, a recent study found that the region in the *Prochlorococcus* tree that separates clades with chitin metabolism from those without it contains unique photophysiology and is linked to a massive expansion of photosystem genes [98]. This is consistent with the photosynthetic machinery of *Prochlorococcus* coming under intense selection during the evolutionary transition to a dedicated planktonic lifestyle.

## 5. Implications of the chitin raft hypothesis for biospheric productivity and Earth’s history

The chitin raft hypothesis provides relevant context to the geologic and genomic records of Precambrian cyanobacteria but leaves open questions and can be tested in several ways. For example, planktonic nitrogen-fixing cyanobacteria likely also colonized the ocean sometime in the late Neoproterozoic or early Phanerozoic [34], and the evolution of their lifestyle has not been fully unravelled. Many marine nitrogen-fixing cyanobacteria exist as part of buoyant colonies (e.g. *Trichodesmium*) [99] or in associations with eukaryotic phytoplankton (e.g. *Richellia* or UCYN-A) [100,101], which helps address the question of how they colonized the ocean in the face of aggregation-inducing stresses. Other groups, including *Crocosphaera watsonii* [102,103], have a unicellular planktonic lifestyle, but it is not yet clear if they have unrecognized abilities to switch between planktonic and attached lifestyles as we recently discovered in marine picocyanobacteria. Further studies on the diversity of lifestyles and lifestyle-associated traits among nitrogen-fixing marine cyanobacteria as well as studies on when in time different groups diverged from each other, as calculated by molecular clocks, will test, or at least provide nuance to, the chitin raft hypothesis.

In terms of the geologic record, additional targeted searches for evidence of planktonic cells in Proterozoic sediments, either from fossils of smaller cells [4–6] or from biomarkers [53], especially from any deep-water depositional environments, would provide important tests that could invalidate our proposal. Further examination of nearshore-to-offshore gradients in carbon isotopes can in turn provide indirect evidence of the presence and productivity of potential planktonic cyanobacteria in open ocean waters. If high CO_2_ levels muted such signals in the Archaean and early Proterozoic [46,47], then one might expect such gradients to become stronger in the later Proterozoic when CO_2_ levels are expected to have been somewhat lower [48–50], as is potentially seen in some late-Mesoproterozoic sediments [104]. Increasing the number of available records to improve statistics and extending samples to include further-offshore environments less likely to be coupled to continental shelf environments through physical transport processes will help understand whether there was significant organic carbon production in the photic zone of the deep open ocean, potentially indicative of planktonic cyanobacteria.

**Figure 3.**
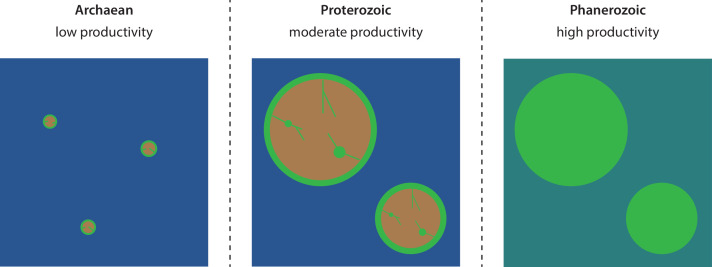
Proposed model for the two-stage evolution of biospheric productivity over Earth’s history. The emergence of large sub-aerial continents near the Archaean–Proterozoic boundary drove a first major increase in biospheric productivity due to primary production being largely restricted to continental shelves. Colonization of the oceans by marine picocyanobacteria and eukaryotic algae and colonization of the continents by land plants drove a second major increase in productivity near the Proterozoic–Phanerozoic boundary. Notes: green circumferences of brown landmasses represent continental shelf environments where productivity is inferred to have been localized during the Precambrian, whereas green lines/circles within terrestrial environments represent rivers and lakes. Greening of oceans and continents in the Phanerozoic represent the global spread of chlorophyll and thus of oxygenic photosynthesis.

If the chitin raft hypothesis holds up in the face of additional scrutiny, it has implications for interpreting large-scale patterns of biospheric evolution and atmospheric oxygenation over Earth’s history. That is, if oxygenic photosynthesis was largely restricted to shallow continental shelf environments during the Precambrian, then the total productivity of the marine biosphere would depend on the total global volume of benthic habitats as well as the supply of nutrients to those habitats (figure 3). It has in this context become increasingly clear from geochemical studies and geophysical models that there was a major increase in sub-aerial continental surface area between the late Archaean and early Proterozoic [37,105–108]. While evidence suggests that the emergence of continents above water extends back at least to the Paleoarchean (3.6–3.2 Ga) [109,110], continents at that time were likely small, with a total emerged mass reaching perhaps as low as only a few percent of modern continents [105–108]. In contrast, the aerial extent of emerged continents is thought to have reached near-modern levels at the start of the Proterozoic [106], in turn greatly increasing their weatherability [106,107]. Hence, a transition from a ‘water world’ to a world with large, exposed continents would have led to major expansions in both the global extent of benthic habitats and the weathering supply of nutrients to those habitats. If oxygenic photosynthesis was restricted to continental shelves during this period, as the chitin raft hypothesis suggests, these combined effects would have led to a major increase in primary production [43], possibly helping fuel the Great Oxidation Event (GOE) [16–18].

Evidence from molecular clocks and fossils of cyanobacterial evolution provides additional context and support to these ideas. That is, calculations suggest an increased rate in the colonization of freshwater environments by cyanobacteria across the late Archaean and early Proterozoic [65], consistent with the expansion of freshwater habitats associated with the emergence of large continents [106,107]. Molecular clocks also indicate increased diversification of cyanobacterial morphologies during this period, including the emergence of various filamentous forms capable of forming highly productive dense mats [111]. Indeed, combined results from fossils [58] and clocks [8] suggest the emergence of thylakoid-bearing cyanobacteria near the Archaean–Proterozoic boundary. These findings are all consistent with both expanded ecological opportunities for cyanobacteria in benthic environments and increasing productivity of benthic ecosystems associated with the rise of continents in the lead up to the GOE.

It has been proposed that terrestrial cyanobacteria were major contributors to Precambrian biospheric productivity, with cyanobacteria in soil crusts responsible for a large fraction thereof [112]. Microbial mats with geochemical signatures suggestive of cyanobacterial photosynthesis have been identified in riverbank deposits as old as approximately 3.2 Ga [113], broadly consistent with these views. However, soil crust cyanobacteria require liquid water to become metabolically active [114], and modelling studies suggest that continental interiors were substantially drier before the Phanerozoic rise of land plants due to the role that their evapotranspiration plays in trapping and recycling water on land [115]. Productivity of terrestrial cyanobacteria was therefore likely mostly restricted to rivers and lakes during the Proterozoic. Indeed, including estimates of soil moisture distributions into account considerably lowers modelled productivity of Precambrian terrestrial soil crust ecosystems [112]. Moreover, even though extant terrestrial cyanobacteria have evolved effective mechanisms for protection from UV radiation [114], even moderate exposure to UV dramatically lowers the productivity of mats [116], and UV levels were likely higher in the Proterozoic due to a less-developed ozone layer [86]. However, it has also been argued that dry Precambrian continents would have destabilized the carbon cycle due to insufficient silicate weathering, leading to increased CO_2_ levels, higher temperatures and increased rainfall [117], thereby potentially boosting productivity of soil crust cyanobacteria [112]. The balance of these different effects and their combined impact on the productivity of terrestrial cyanobacteria is unclear and would benefit from further study. Still, regardless of the balance of productivity between benthic and terrestrial ecosystems, in the absence of oxygenic photosynthesis in the open ocean, the rise of continents near the Archaean–Proterozoic boundary would have greatly increased total biospheric productivity (figure 3).

In closing, the chitin raft hypothesis offers possible insights into the broader Neoproterozoic–Phanerozoic biospheric revolutions and atmospheric oxygenation that ultimately led to the rise of modern marine ecosystems. Genomic and fossil evidence highlight an increased diversification of eukaryotes over the second half of the Proterozoic [7,15,54,55,77,118], eventually leading to the rise of eukaryotic algae in the late Neoproterozoic [119], perhaps partly due to increased availability of phosphorus in the aftermath of Snowball Earth glaciations [119]. The presence of eukaryotic algae likely contributed to increased productivity in benthic environments [3], in turn making more food available to higher trophic levels, thereby playing a key role in the diversification of animals [15,119]. Among the newly emerging groups were diverse arthropods that evolved armoured exoskeletons made of chitin both for defence against predators [15] and to improve their ability to prey on other groups [44]. The by-product of the evolution of chitin-based exoskeletons in arthropods was that it created surfaces where picocyanobacteria could aggregate to avoid environmental stresses in the water column (figure 2), creating for the first time a route to begin exploring the open ocean. Finally, the chitin raft hypothesis suggests these events gave way to a macroevolutionary dynamic in which co-expanding populations of arthropods and marine picocyanobacteria both created increasing ecological opportunities for each other. This potentially self-reinforcing dynamic would represent a kind of collective ‘niche construction’ [120] in open ocean waters that we argue helped seed the rise of modern marine ecosystems [67]. In this view, the emergence of pelagic planktonic marine cyanobacteria reflects the broader biospheric expansion into the open ocean, which was not possible until ecosystems reached sufficient levels of complexity.

## Data Availability

This article has no additional data.
